# Effect of Delayed-Release and Extended-Release Methylphenidate on Caregiver Strain and Validation of Psychometric Properties of the Caregiver Strain Questionnaire: Results from a Phase 3 Trial in Children with Attention-Deficit/Hyperactivity Disorder

**DOI:** 10.1089/cap.2020.0159

**Published:** 2021-04-16

**Authors:** Frank A. López, Stephen V. Faraone, Jeffrey H. Newcorn, Helen A. Doll, Stephanie Rhoten, Hannah B. Lewis, Tayyaba F. Khan, Norberto J. DeSousa, Floyd R. Sallee, Bev Incledon

**Affiliations:** ^1^ Pediatric Neurology, P.A., Winter Park, Florida, USA.; ^2^ Departments of Psychiatry and of Neuroscience and Physiology, State University of New York (SUNY) Upstate Medical University, Syracuse, New York, USA.; ^3^ Mount Sinai Medical Center, New York, New York, USA.; ^4^ Clinical Outcomes Assessments, ICON plc, Oxford, United Kingdom.; ^5^ Patient Centered Outcomes, ICON plc, South San Francisco, California, USA.; ^6^ Patient Centred Outcomes, ICON plc, London, United Kingdom.; ^7^ Highland Therapeutics, Inc., Toronto, Canada.; ^8^ Ironshore Pharmaceuticals & Development, Inc., Camana Bay, Grand Cayman, Cayman Islands.; ^9^ Ironshore Pharmaceuticals, Inc., Durham, North Carolina, USA.; ^†^ Current address: IQVIA, San Francisco, California, USA.

**Keywords:** DR/ER-MPH, caregiver strain, HLD200, methylphenidate, psychometrics, validation

## Abstract

***Objectives:*** Inadequately controlled symptoms and associated impaired functioning have a significant negative impact on caregivers of children with attention-deficit/hyperactivity disorder (ADHD). This study aimed to assess the impact of evening-dosed, delayed-release and extended-release methylphenidate (DR/ER-MPH) treatment on caregiver strain, measured by the Caregiver Strain Questionnaire (CGSQ), and present *post hoc* psychometric analyses assessing the reliability and validity of the CGSQ, its ability to detect change (responsiveness), and to derive responder definitions.

***Methods:*** The CGSQ was an exploratory efficacy endpoint in a phase 3, 3-week, randomized, double-blind, multicenter, placebo-controlled, forced-dose titration trial of DR/ER-MPH in children aged 6–12 years with ADHD (NCT02520388). Psychometric properties of the CGSQ evaluated *post hoc* included internal consistency using Cronbach's alpha; test/retest reliability using intraclass correlation coefficients (ICCs); construct validity (known groups and convergent/divergent validity); responsiveness to changes in assessments of ADHD severity (ADHD Rating Scale-IV [ADHD-RS-IV], Conners' Global Index–Parent [CGI-P], and Clinical Global Impression—Severity [CGI-S]/CGI—Improvement [CGI-I]); and meaningful change threshold (MCT) using receiver operating characteristic curves, which were used to compare response between DR/ER-MPH and placebo groups.

***Results:*** Randomized DR/ER-MPH (54.5) and placebo (54.9) groups had similar mean CGSQ scores at screening. Caregivers of children on DR/ER-MPH reported significant reductions in CGSQ scores after 3 weeks of DR/ER-MPH treatment versus placebo (least-squares mean: 41.2 vs. 49.1; *p* < 0.001). The CGSQ demonstrated strong internal consistency (Cronbach's alpha = 0.93) and good test/retest reliability (ICC = 0.72). Known groups, convergent/divergent validity, and responsiveness were demonstrated from relationships between the CGSQ and the CGI-S, ADHD-RS-IV, and CGI-P. The mean anchor-based MCT for CGSQ total score was estimated as −9.0 (DR/ER-MPH vs. placebo: 53.2% vs. 29.9% *p* = 0.003).

***Conclusions:*** CGSQ scores significantly decreased after 3 weeks of DR/ER-MPH treatment versus placebo, and the CGSQ was found to be a valid and reliable measure of strain in caregivers of children with ADHD. Clinical trial registration identification number: NCT02520388.

## Introduction

Attention-deficit/hyperactivity disorder (ADHD) is a chronic neurodevelopmental disorder characterized by symptoms of inattention and/or hyperactivity/impulsivity and impaired functioning (American Psychiatric Association 2013; Sallee 2015). Individuals with ADHD experience symptoms and associated impairments from the time of awakening until bedtime. Long-acting stimulants, including methylphenidate (MPH), are recommended as first-line treatment for children and adolescents with ADHD (Pliszka et al. 2017). Although commonly prescribed extended-release stimulants are effective, there remains an unmet need for control of ADHD symptoms and functional impairment that lasts throughout the entire waking day (Whalen et al. 2006; Sallee 2015; Childress 2016; Faraone et al. 2017).

Inadequate control of ADHD symptoms throughout the entire waking day not only impacts the affected individual but also leads to adverse outcomes for families and caregivers. For working parents, most interactions with their children typically occur in the mornings and evenings. Several studies have described that inadequate control of symptoms and related impairment during these bookends of the day is a source of strain for families and caregivers of children with ADHD that persists despite treatment with ADHD medications (Coghill et al. 2008; Sallee 2015; Faraone et al. 2017). In this article, and as previously described (Brannan et al. 1997), caregiver strain refers to the demands, difficulties, and negative consequences experienced by caregivers of children with mental, emotional, and behavioral problems.

In many studies, parents of children with ADHD have identified their children's behavior as their primary source of stress and these burdens persist despite the use of medications (Brown and Pacini 1989; Coghill et al. 2008; Fridman et al. 2017; Leitch et al. 2019). Caregiver strain increases with increasing ADHD symptom severity, and the effect of symptom severity on treatment seeking is often mediated by caregiver burden (Angold et al. 1998; Theule et al. 2013; Bussing et al. 2015; Babinski et al. 2019). Very few studies, however, have investigated the effect of ADHD medication on caregiver strain. Studies have reported the positive impact of medication on caregivers and/or family using instruments such as the Family Strain Index or quality-of-life measurements (Svanborg et al. 2009; Kim et al. 2014; Silva et al. 2015), but they have not specifically measured the strain that caregivers attribute to caring for a child with ADHD.

Child/parent interactions have been identified as a key contributing factor to strain in caregivers of children with mental health conditions (Frank et al. 2017). Studies have reported improvements in child/parent interactions or child/parent conflicts with ADHD medication (Wilens et al. 2006); however, whether the duration of medication effect into the evening improves child/parent interactions remains equivocal. Stein et al. (1996) reported a reduction in parent/child conflicts with both twice-daily (BID) and thrice-daily (TID) stimulant treatment versus placebo; however, there was no significant difference between BID and TID conditions despite improved evening behavior with TID administration. On the contrary, Chronis et al. (2003) reported that parents reported improved pleasantness in interactions with their children receiving an afternoon stimulant dose versus placebo.

An instrument that focuses specifically on the added strain of caring for a child with an emotional or behavioral disorder is the Caregiver Strain Questionnaire (CGSQ), which covers several areas of caregiver strain. It was originally developed to measure the strain experienced by caregivers of children with serious emotional and behavioral problems over a previous 6-month period. It was validated initially in a sample of caregivers of children with parents in the military who needed mental health services, with a reported internal consistency (Cronbach's alpha) of 0.93 (Brannan et al. 1997). The CGSQ has since been reported as a valid and reliable instrument across a variety of caregiver samples, including caregivers of children with autism (Stuart and McGrew 2009; Khanna et al. 2012), and Medicaid-enrolled children with emotional and behavioral disorders (Taylor-Richardson et al. 2006). For caregivers of children with ADHD, only the reliability of the CGSQ has been reported (Cronbach's alpha = 0.92; Vander Stoep et al. 2017).

There is a lack of psychometric data concerning the validity of the CGSQ in caregivers of children with ADHD who are receiving pharmacotherapy. The work described in this article provides psychometric data for the CGSQ in a sample of caregivers of children with ADHD treated with delayed-release and extended-release MPH (DR/ER-MPH; trade name: JORNAY PM^®^). DR/ER-MPH (formerly HLD200) is an evening-dosed ADHD medication designed to delay the initial release of MPH by ∼8 to 10 hours to provide onset of treatment effect upon awakening, lasting into the evening (Childress et al. 2018). Furthermore, without an immediate-release component, DR/ER-MPH has a monophasic pharmacokinetic profile without multiple peaks or troughs during the day (Childress et al. 2018). Two pivotal phase 3 trials of children with ADHD uniquely demonstrated significant improvements in ADHD symptoms and reduced functional impairment from awakening to evening with DR/ER-MPH treatment compared with placebo (Pliszka et al. 2017; Childress et al. 2020).

Herein, we present a prespecified exploratory efficacy endpoint from one of the phase 3 trials (ClinicalTrials.gov identifier: NCT02520388) that assessed the effects of DR/ER-MPH on caregiver strain, as measured by the CGSQ (Brannan et al. 1997), after 3 weeks of treatment with DR/ER-MPH. *Post hoc* psychometric analyses of the CGSQ are also presented here to confirm the choice of the scale as an adequate measure of strain and to support the validity of treatment effects seen in the phase 3 trial.

## Methods

### Study participants

Male and female children aged 6–12 years with ADHD were enrolled if they met the predefined study inclusion and exclusion criteria. Key inclusion criteria included the following: a diagnosis of ADHD based on the *Diagnostic and Statistical Manual of Mental Disorders, 5th edition* (DSM-5); ADHD Rating Scale-IV (ADHD-RS-IV) score ≥90th percentile for age and gender, and ≥26, at baseline; Clinical Global Impression—Severity (CGI-S) score ≥4 and Conners' Global Index—Parent (CGI-P) score >10 at baseline; at least a partial clinical response to MPH by judgment of the investigator; and early morning functional impairment and/or difficulties performing a morning routine of ≥30 minutes between 6:00 AM and 9:00 AM. Key exclusion criteria included the following: history of or current medical condition or laboratory result that could either jeopardize participant safety or interfere with study participation; history of psychosis, bipolar disorder, anorexia nervosa, bulimia, or suicide attempt; current depression, anxiety, conduct disorder, substance use disorder, or other psychiatric conditions; history of severe allergic reaction or intolerance to MPH; and past use of psychotropic medications, including antidepressants, mood stabilizers, and antipsychotics (with the exception of stimulants and nonstimulants for the treatment of ADHD).

### Study design

The study was conducted in two phases: a screening/washout phase lasting up to 2 weeks (washout of ≥72 hours before randomization), and a 3-week, randomized, placebo-controlled phase utilizing a forced-dose titration schedule. At the start of the treatment phase, participants were randomized in a 1:1 ratio to receive either DR/ER-MPH or placebo once daily each evening for 3 weeks. Dosing was initiated at 40 mg/day each evening at 8:00 PM (±30 minutes) for 1 week, with scheduled titration, as tolerated, over the subsequent 2 weeks to 60 and 80 mg/day. The maximum allowable dose was 3.7 mg/kg/day and a 20-mg decrement was permitted for safety or tolerability reasons. Participants unable to tolerate at least 40 mg/day during the final week were discontinued. Participants were also permitted to adjust the evening dosing time between 6:30 PM and 9:30 PM in 30- or 60-minute increments per week to achieve optimal morning control of observed ADHD symptoms.

As previously reported (Pliszka et al. 2017), the study was conducted in accordance with Good Clinical Practice guidelines and the Declaration of Helsinki. All participants and parents/legal guardians provided informed assent and consent, respectively, under procedures approved by each study site's institutional review board.

### Assessments

The CGSQ score at week 3 was prespecified as an exploratory efficacy endpoint to determine whether DR/ER-MPH improves caregiver strain. The CGSQ is a 21-item caregiver-reported questionnaire assessing strain experienced by caregivers caring for a child with emotional and behavioral challenges (Brannan et al. 1997). It consists of three subscales: objective strain, subjective externalized strain, and subjective internalized strain. Each item is rated on a 5-point scale, from 1 denoting “not at all” to 5 denoting “very much.” A CGSQ total score was calculated by summing the responses on each item of the scale, with total possible scores ranging from 21 to 105.

The CGSQ was originally developed to evaluate caregiver strain over a preceding 6-month period (Brannan et al. 1997). In this trial, the recall period was revised so that the CGSQ evaluated caregiver strain over a preceding 3-week period. This was the only revision made to the original CGSQ. Due to the 3-week recall period, the CGSQ was administered at screening (i.e., before washout of prior ADHD therapy) and following the 3-week treatment phase (week 3) to allow for assessment of caregiver strain while children were on a consistent therapeutic regimen. Therefore, comparisons were only made between DR/ER-MPH and screening, before washout of previous medication if necessary, not a purely untreated baseline. Mean change in CGSQ score was calculated between screening and the end of the 3-week treatment phase.

All efforts were made to maintain the same parent/guardian rater for each individual participant. Higher scores on all the CGSQ items indicate greater strain, except for the item “How well did you relate to your child?” which was reverse coded so that a higher score indicates greater strain. Reductions in total CGSQ scores indicate an improvement.

The psychometric evaluation of CGSQ total scores used data from several scales: the ADHD-RS-IV, CGI-P, CGI-S, and Clinical Global Impression—Improvement (CGI-I). The ADHD-RS-IV measures severity of ADHD symptoms, with 18 items rated on a 4-point scale from 0 (reflecting no symptoms) to 3 (reflecting severe symptoms), and total possible scores ranging from 0 to 54 (DuPaul et al. 1998). Psychometric analyses used ADHD-RS-IV data from screening and week 3. Ratings on the ADHD-RS-IV were also obtained specifically from 6:00 AM to 9:00 AM (ADHD-AM-RS). Psychometric analyses used ADHD-AM-RS data rated over the previous week from baseline (randomization) and week 3. Higher scores indicate greater symptom severity.

The CGI-P is a 10-item caregiver-completed rating scale designed to assess features of general psychological difficulty that may be expressed behaviorally, academically, socially, or emotionally in children and adolescents aged 6 to 18 years (Conners 1989). It consists of two subscales: restless-impulsive and emotional lability. Each item is rated on a 4-point scale, with 0 denoting “never/seldom” and 3 denoting “very often/frequently.” Psychometric analyses used CGI-P data from screening, which assessed the previous month, and week 3 assessing the previous week. Reductions in CGI-P scores indicate an improvement.

Finally, the CGI-S and CGI-I are clinician-rated global scales that provide assessment of severity (CGI-S) and its change from baseline (CGI-I). For the psychometric analyses, the CGI-S scores from screening were used. It is rated on a 7-point scale from 1 (“normal”) to 7 (“extremely ill”). The CGI-I was administered at week 3 and is also rated on a 7-point scale from 1 denoting “very much improved” to 7 denoting “very much worse” (Guy 1976). Scores below the midpoint indicate improvement and scores above the midpoint indicate worsening.

### Statistical and psychometric analyses

All analyses were performed on the intention-to-treat (ITT) population, defined as all randomized participants who received at least one dose of study drug and had at least one postbaseline evaluation on the ADHD-RS-IV. The effect of DR/ER-MPH treatment on the CGSQ compared with placebo was assessed at week 3 using a prespecified analysis of covariance model with treatment as the main effect, and study center and baseline score as the covariates.

The *post hoc* psychometric analyses were conducted as part of the validation process for the CGSQ as a measurement of caregiver strain. Internal consistency for caregivers of children with ADHD was evaluated at screening using Spearman correlations and Cronbach's alpha. Spearman correlations of each item with the total score, omitting that item, were calculated. A threshold of 0.30 (Stevens 1951) was used to evaluate the Spearman correlations. Cronbach's alpha values were calculated for all items and after each item was deleted.

Test/retest reliability was evaluated using intraclass correlation coefficients (ICCs) between CGSQ total scores at screening and week 3. Test/retest reliability is the degree to which scores on a scale are consistent when an individual is evaluated under the same conditions, but on different occasions. Ideally, every variable needs to be kept the same, so that the scale can only reflect the construct it is designed to measure (Aldridge et al. 2017). As the evaluation of test/retest reliability of the CGSQ requires assessment of participants under identical treatment conditions, which the study protocol did not account for, assessments were done in participants considered stable, (i.e., those having minimal ADHD-RS-IV score changes of −3 to +3 from screening to week 3). A two-way mixed ICC for absolute agreement was used to assess test/retest reliability (Shrout and Fleiss 1979). While there are no widely agreed upon benchmarks that can be used in the interpretation of the ICC, scale-level ICCs of ≥0.80 (Nunnally and Bernstein 1994) have been proposed as acceptable. For the purposes of this study, the following scheme was used: ICC <0.60 = poor; 0.60 to 0.69 = moderate; 0.70 to 0.79 = good; and 0.80 to 1.0 = very good.

Known-groups validity of the CGSQ was assessed by comparing CGSQ total scores at screening between subgroups defined by measures of ADHD severity (i.e., scores on the ADHD-RS-IV, CGI-S, and CGI-P grouped by tertiles). Convergent and divergent validity refers to the extent to which a measure relates to other measures or variables based on theoretical content, or the expected relationship with the variable(s) chosen. The CGSQ was expected to be correlated with both the ADHD-RS-IV and ADHD-AM-RS because the strain on the caregiver is presumed to be related to the severity of the ADHD symptoms (Angold et al. 1998).

Convergent and divergent validity analyses were based on the hypothesis that CGSQ total scores would be more strongly related to ADHD-RS-IV than ADHD-AM-RS, given that the former assesses outcomes throughout the day, as does the CGSQ. The ADHD-AM-RS assesses morning symptoms only and thus was expected to be less strongly associated with the CGSQ.

The ability of the CGSQ to detect change was evaluated by computing Spearman rank correlations between changes in CGSQ total scores and changes in the CGI-P, ADHD-RS-IV, ADHD-AM-RS, and CGI-I scores. Mean change in CGSQ scores were also compared between participants categorized into tertiles (based on direction and response on CGI-P, ADHD-RS-IV, ADHD-AM-RS, and CGI-I) and among participants categorized into predefined ADHD-RS-IV change categories (worse, stable, minimal improvement, and much improvement in ADHD-RS-IV scores from screening to week 3). Statistical significance was determined using ANOVAs with tests for linear trend to determine if mean changes in scores were different between groups.

Anchor-based estimates of meaningful change (meaningful change threshold [MCT]) for the CGSQ were identified to help interpret CGSQ total scores by providing responder definitions. Receiver operating characteristic curves were used to estimate MCTs by identifying the CGSQ score changes that best discriminated those simultaneously reaching two well-accepted response criteria (≥30% decrease in ADHD-RS-IV and patients categorized into CGI-I of 1 or 2) from those who do not (<30% decrease and CGI-I >2). A mean MCT, based on the responder definitions anchored to ADHD-RS-IV and CGI-I, was calculated and used as a responder definition to compare response (the proportion of patients reaching the responder definition) between DR/ER-MPH and placebo treatment groups using chi-square statistics.

## Results

### Baseline characteristics

**FIG. 1.**
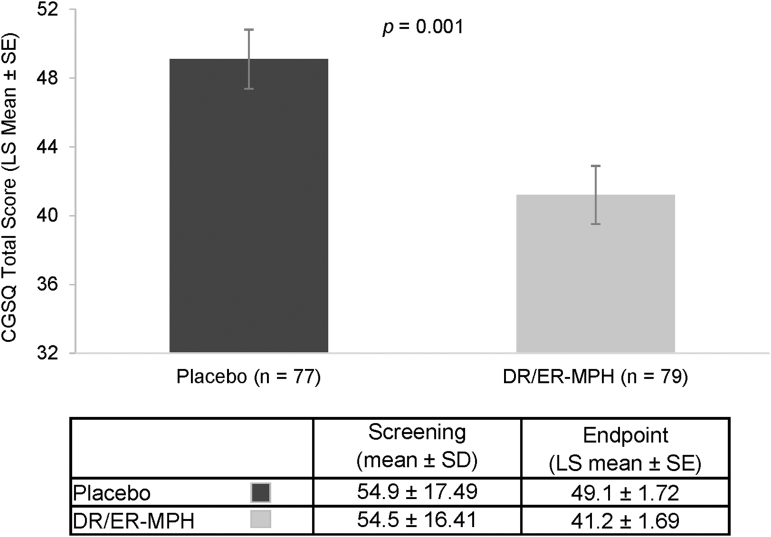
CGSQ total scores after 3 weeks of treatment. Error bars denote standard error. CGSQ, Caregiver Strain Questionnaire; DR/ER-MPH, delayed-release and extended-release methylphenidate; LS, least-squares; SD, standard deviation; SE, standard error.

Of the 163 children enrolled across 22 sites, a total of 161 children were included in the ITT population (DR/ER-MPH, *n* = 81; placebo, *n* = 80). Demographics and baseline characteristics of the study population have been described in detail elsewhere and were comparable between treatment groups (Pliszka et al. 2017). The mean final dose of DR/ER-MPH was 68.1 mg and ranged from 40 to 80 mg, and the most commonly prescribed final dosing time was 8:00 PM (83.8%) and ranged from 7:00 PM to 9:00 PM (Pliszka et al. 2017). When the baseline characteristics of randomized participants were evaluated, there were no significant differences in mean CGSQ total scores at screening between treatment groups (54.5 vs. 54.9; Fig. 1). At screening, 60.2% of participants were taking at least one medication for ADHD, with 58.4% of participants taking a stimulant medication and 9.3% taking a nonstimulant medication. The proportion of participants using each of these classes of prior medications was similar in the DR/ER-MPH and placebo groups.

### Effect of DR/ER-MPH on caregiver strain

After 3 weeks of treatment, caregivers of children treated with DR/ER-MPH reported significantly lower strain compared with caregivers of children who received placebo, as measured by CGSQ total scores (least-squares means: 41.2 vs. 49.1; *p* = 0.001; Fig. 1). Mean CGSQ score decreased 12 points (20.6%) from screening to week 3 in the DR/ER-MPH group compared with a 4-point decrease (4.4%) in the placebo group (*p* < 0.001). When evaluated by randomized groups, mean CGSQ scores at screening were comparable between DR/ER-MPH and placebo.

### Psychometric properties of the CGSQ

#### Reliability

Internal consistency of the CGSQ at screening was strong (Cronbach's alpha = 0.93). Cronbach's alpha remained the same when each item was deleted, and each item-total correlation was above the threshold of 0.30 (Stevens 1951), suggesting that all items contribute equally to the measure (Table 1). The CGSQ also demonstrated good test/retest reliability (ICC = 0.72; Table 2) among caregivers of stable participants (i.e., those with ADHD-RS-IV score changes between −3 and +3 from screening to week 3). Participants who were characterized as stable (*n* = 31) had no statistically significant change in mean (standard deviation) ADHD-RS-IV scores from screening (30.1 [10.4]) to week 3 (38.9 [11.4]).

**Table 1.** Reliability of the Caregiver Strain Questionnaire

CGSQ item	Cronbach's alpha	Item-total correlations^a^
1. Interruption of personal time	0.93	0.72
2. Missing work	0.93	0.71
3. Family routine disruption	0.93	0.72
4. Having to do without things	0.93	0.71
5. Negative mental/physical health effects	0.93	0.68
6. Child getting into trouble	0.93	0.57
7. Financial strain	0.93	0.58
8. Less attention to other family members	0.93	0.72
9. Disruption of family relationships	0.93	0.78
10. Disruption of family social activities	0.93	0.73
11. Feeling isolated	0.93	0.73
12. Sad or unhappy	0.93	0.72
13. Embarrassed	0.93	0.66
14. Relating to child (reversed score)	0.94	0.32
15. Feeling angry toward child	0.93	0.46
16. Worried about child's future	0.93	0.51
17. Worried about family's future	0.93	0.58
18. Guilty about child's illness	0.93	0.58
19. Resentful	0.93	0.51
20. Tired or strained	0.93	0.70
21. Toll taken on family	0.93	0.79

^a^ Item-total correlations calculated using Spearman's rank correlation coefficient.

CGSQ, Caregiver Strain Questionnaire.

**Table 2.** Reliability, Validity, and Responsiveness of the Caregiver Strain Questionnaire

Psychometric property	CGSQ total
Reliability	
Cronbach's α	0.93
ICC (95% CI)	0.72 (0.50–0.86)
Validity^a^	
ADHD-RS-IV	0.457 (*p* < 0.0001)
ADHD-AM-RS^b^	0.265 (*p* = 0.0006)
Sensitivity to change^a^	
ADHD-RS-IV	0.62 (*p* < 0.0001)
ADHD-AM-RS^b^	0.54 (*p* < 0.0001)
CGI-P	0.65 (*p* < 0.0001)
CGI-I	0.61 (*p* < 0.0001)

^a^ Spearman's rank correlations.

^b^ ADHD-AM-RS at baseline.

ADHD-AM-RS, attention-deficit/hyperactivity disorder rating scale-IV for 6:00 AM to 9:00 AM only; ADHD-RS-IV, attention-deficit/hyperactivity disorder rating scale-IV; CGI-I, Clinician Global Impression—Improvement; CGI-P, Conners' Global Index—Parent; CGSQ, Caregiver Strain Questionnaire; ICC, intraclass correlation coefficient.

### Construct validity

Known groups validity was demonstrated for the CGSQ across all groups of increasing ADHD severity. CGSQ total scores increased linearly with CGI-S categories (*p* < 0.05) as well as ADHD-RS-IV (*p* < 0.0001) and CGI-P (*p* < 0.0001) scores. Mean CGSQ total scores were also progressively higher (worse) as ADHD severity worsened (as measured by CGI-S, CGI-P, and ADHD-RS-IV tertiles), with significant linear trends (*p* < 0.0001; Supplementary Table S1).

Convergent and divergent validity of CGSQ total scores was assessed using correlations with both ADHD-RS-IV and ADHD-AM-RS scores at screening and baseline, respectively. As hypothesized, the correlation was stronger between CGSQ total scores and ADHD-RS-IV (Spearman correlation: 0.457, *p* < 0.0001) than with ADHD-AM-RS (0.265, *p* = 0.0006) confirming convergent and divergent validity of the CGSQ (Table 2).

### Ability to detect change

Mean changes in CGSQ total scores were found to have significant linear trends (*p* = 0.004) across ADHD-RS-IV change categories, suggesting that improvements in ADHD-RS-IV scores are reliably related to improvements in CGSQ total scores (Supplementary Table S2). There were also significant positive correlations (Spearman correlations: 0.54–0.65, all *p* < 0.0001) between changes in CGSQ total scores with changes in ADHD-RS-IV, ADHD-AM-RS, and CGI-P scores from screening to week 3 (baseline to week 3 for ADHD-AM-RS) as well as with CGI-I absolute scores at week 3 (Table 2). When changes in CGSQ total scores were examined within the tertiles of ADHD severity, greater changes in ADHD-RS-IV, ADHD-AM-RS, and CGI-P change scores were associated with significantly greater changes in CGSQ total scores (all *p* < 0.0001). Greater improvement reported in CGI-I ratings was also associated with greater improvements (reductions) in CGSQ total scores (all *p* < 0.0001; Supplementary Table S2).

### Interpretation of CGSQ scores

The mean anchor-based MCT for CGSQ total score was estimated as −9.0, computed as an average of the MCTs for ≥30% decrease in ADHD-RS-IV (−6), and CGI-I of 1 or 2 (−12). On using this MCT estimate as a responder definition, the proportion of participants with a meaningful change was significantly greater among the DR/ER-MPH treatment group versus placebo (53.2% vs. 29.9% *p* = 0.003; Table 3), which corresponds to a number needed to treat statistic of 4.3. This indicates that only about four pediatric patients with ADHD need to be treated to achieve a clinically meaningful outcome on the CGSQ compared with placebo.

**Table 3.** Proportion of Participants Reaching the Responder Definition from Screening to Week 3

	Responder definition (−9)^a^
DR/ER-MPH, *n* (%)	42 (53.2)
Placebo, *n* (%)	23 (29.9)
Difference (%)	23.3
95% confidence interval	8.0–38.5
*p-*Value^b^	0.003

^a^ Responder definition was estimated as the mean MCT for the following anchors: ≥30% decrease in ADHD-RS-IV (−6) and CGI-I ≤2 (−12).

^b^ *p*-Value from chi-square test.

ADHD-RS-IV, attention-deficit/hyperactivity disorder rating scale-IV; CGI-I, Clinician Global Impression—Improvement; DR/ER-MPH, delayed-release and extended-release methylphenidate; MCT, meaningful change threshold.

## Discussion

This study reports a prespecified exploratory efficacy endpoint from a randomized-controlled trial assessing the efficacy of DR/ER-MPH compared with placebo in children aged 6 to 12 years with ADHD. Improvements in mean CGSQ score demonstrated a statistically significant reduction in strain experienced by caregivers during the 3 weeks of treatment with DR/ER-MPH compared with placebo. *Post hoc* analyses also demonstrated that a significantly greater proportion of participants receiving DR/ER-MPH achieved a clinically meaningful improvement on the CGSQ compared with those receiving placebo. Additionally, *post hoc* psychometric analyses support the use of the CGSQ to measure reliably and validly the strain experienced by caregivers of children with ADHD.

In this study, 3 weeks of DR/ER-MPH treatment decreased the severity of caregiver strain, as demonstrated by lower CGSQ scores, despite 60.2% of children being treated for ADHD at screening. We postulate that improved CGSQ scores after 3 weeks of DR/ER-MPH treatment are related to improved control of ADHD symptoms and functional impairment over an extended duration of the day, including the early morning and late afternoon/evening (Pliszka et al. 2017; Childress et al. 2020). The prior equivocal findings regarding extended treatment effect into the evening and improvement of child/parent interactions (Stein et al. 1996; Chronis et al. 2003) suggest that a full-day duration of treatment effect, including the early morning, may be an important factor in improving child/parent interactions and reducing the extent of caregiver strain.

The results from the present study suggest that assessing symptoms and functional impairment at the bookends of the day using validated rating scales such as the Before School Functioning Questionnaire and the Parent Rating of Evening and Morning Behavior, Revised (PREMB-R) morning and evening subscales (Sutton et al. 2003; Faraone et al. 2018a, 2018b) and targeting interventions to address early mornings and evenings may be key to reducing caregiver strain.

This is the first time the CGSQ has been validated to measure caregiver strain over a preceding 3-week period; the original scale was developed to measure caregiver strain over the past 6 months (Brannan et al. 1997). The CGSQ used here, with a shorter recall, is therefore suitable for use in clinical intervention trials of pediatric ADHD as a treatment outcome. The CGSQ may also be useful in clinical practice to identify the challenges of families shortly after treatment initiation and/or switches in medication. Previous research has also identified the CGSQ as a clinically relevant, brief, no-cost measure that is easily accessible, providing support for its clinical utility (Holly et al. 2019).

The toll taken on families and caregivers is often what prompts caregivers to seek treatment for their child (Angold et al. 1998). If a medication improves ADHD symptoms in children but caregivers continue to experience strain, this may affect the continuity of treatment or caregiver satisfaction with treatment (Bauer et al. 2019). This might be especially important for parents with ADHD, as the burden of caring for a child with ADHD is even more exacerbated (Chronis-Tuscano et al. 2008). This is the first study to use the CGSQ as a measurement of pharmacological treatment effect on caregiver strain in parents and guardians of children with ADHD, and supports the psychometric validity of the CGSQ to broaden the possible outcome assessments for ADHD treatment.

The analyses presented in this article have several limitations. First, the effect of DR/ER-MPH on caregiver strain was an exploratory endpoint and should therefore be considered hypothesis generating. Second, potential confounders that may affect caregiver strain, such as the level of social support available to caregivers, were not measured. Significant improvements in caregiver strain were observed with DR/ER-MPH despite over half of participants being treated at screening, although it is unknown whether the doses of prior medications were optimized appropriately. It is likely that the increment of improvement in CGSQ scores would have been wider had baseline scores (after washout of prior medication) been used. The trial was not primarily designed to assess the test/retest reliability of the CGSQ, and the interval of 3 weeks between the two CGSQ assessments was slightly longer than the 2-week interval that is most often recommended (Streiner et al. 2019). Given this increased interval, the ICC determined for this trial may therefore be lower than the true test/retest reliability of the CGSQ.

The results should also be considered in light of limitations of the study design discussed previously (Pliszka et al. 2017). The study included children aged 6–12 years, without significant comorbidities, and with at least a partial response to MPH; therefore, the applicability of these findings to parents of children in other age groups, ADHD profiles, and MPH-naive patients, respectively, is unknown. Lastly, the short duration of the study limits extrapolation of findings over the long term.

## Conclusions

Caregivers of children with ADHD reported significant reductions in caregiver strain after 3 weeks of treatment with DR/ER-MPH versus placebo, as measured by the CGSQ. *Post hoc* psychometric analyses found the CGSQ to have strong internal consistency, good test/retest reliability, known groups validity, convergent and divergent validity, and sensitivity to change among caregivers of children aged 6 to 12 years with ADHD. An MCT of −9.0 was estimated for the CGSQ. The proportion of participants receiving DR/ER-MPH who achieved this MCT for the CGSQ was significantly greater than participants receiving placebo.

## Clinical Significance

Several studies have described the considerable burden associated with caring for children with ADHD, even when they are receiving pharmacological treatment. Improvements in caregiver strain with 3 weeks of DR/ER-MPH treatment is likely related to the extended duration of effect of DR/ER-MPH, which provides control of ADHD symptoms from the early morning through to the evening, typically the times when the majority of family interactions occur. The CGSQ was shown to be a valid and reliable instrument for assessing caregiver strain, and may be helpful in clinical practice to optimize treatment outcomes that may affect the whole family.

## Supplementary Material

Supplemental data

Supplemental data
