# Supplementary material for: Effect of Delayed-Release and Extended-Release Methylphenidate on Caregiver Strain and Validation of Psychometric Properties of the Caregiver Strain Questionnaire: Results from a Phase 3 Trial in Children with Attention-Deficit/Hyperactivity Disorder
Source: J Child Adolesc Psychopharmacol. 2021 Apr 16;31(3):179–86. doi: 10.1089/cap.2020.0159 (PMC8066344; doi:10.1089/cap.2020.0159)
Supplement: Supplemental data [file Supp_TableS1.docx]

**Supplementary Information**

**Supplemental Table 1.** Known Groups Validity of the CGSQ

|  | | n | CGSQ Total Scores Mean (SD)^a^ | *p* value^b^ |
| --- | --- | --- | --- | --- |
| CGI-S | |  |  |  |
|  | Tertile 1 | 62 | 47.40 (15.84) | < 0.0001 |
|  | Tertile 2 | 92 | 56.05 (17.70) |  |
|  | Tertile 3 | 9 | 71.89 (17.53) |  |
| CGI-P | |  |  |  |
|  | Tertile 1 | 51 | 41.00 (12.97) | < 0.0001 |
|  | Tertile 2 | 62 | 54.79 (14.25) |  |
|  | Tertile 3 | 50 | 65.10 (18.15) |  |
| ADHD-RS-IV | |  |  |  |
|  | Tertile 1 | 57 | 44.32 (13.92) | < 0.0001 |
|  | Tertile 2 | 52 | 54.44 (17.33) |  |
|  | Tertile 3 | 54 | 62.70 (17.44) |  |

^a^ CGSQ scores at screening ^b^ ANOVA with linear trend
ADHD-RS-IV, attention-deficit/hyperactivity disorder rating scale-IV; CGI-S, Clinician Global Impression–Severity; CGI-P, Conners’ Global Index –Parent; CGSQ, Caregiver Strain Questionnaire; SD, standard deviation
