# Supplementary material for: Effect of Delayed-Release and Extended-Release Methylphenidate on Caregiver Strain and Validation of Psychometric Properties of the Caregiver Strain Questionnaire: Results from a Phase 3 Trial in Children with Attention-Deficit/Hyperactivity Disorder
Source: J Child Adolesc Psychopharmacol. 2021 Apr 16;31(3):179–86. doi: 10.1089/cap.2020.0159 (PMC8066344; doi:10.1089/cap.2020.0159)
Supplement: Supplemental data [file Supp_TableS2.docx]

**Supplemental Table 2.**

Sensitivity to Change of the CGSQ

|  | | n | CGSQ Total Scores Mean Change (SD)^a^ | *p* value^b^ |
| --- | --- | --- | --- | --- |
| CGI-I | |  |  |  |
|  | Tertile 1 | 32 | –23.47 (17.34) | < 0.0001 |
|  | Tertile 2 | 76 | –10.22 (14.47) |  |
|  | Tertile 3 | 48 | 5.96 (13.66) |  |
| CGI-P | |  |  |  |
|  | Tertile 1 | 51 | –18.96 (17.24) | < 0.0001 |
|  | Tertile 2 | 55 | –9.95 (14.28) |  |
|  | Tertile 3 | 49 | 6.61 (11.70) |  |
| ADHD-RS-IV | |  |  |  |
|  | Tertile 1 | 53 | –20.04 (17.12) | <0.0001 |
|  | Tertile 2 | 51 | –8.45 (13.45) |  |
|  | Tertile 3 | 52 | 4.83 (14.44) |  |
| ADHD-AM-RS | |  |  |  |
|  | Tertile 1 | 53 | –18.02 (18.39) | < 0.0001 |
|  | Tertile 2 | 51 | –9.45 (12.19) |  |
|  | Tertile 3 | 52 | 3.75 (16.30) |  |
| ADHD-RS-IV Category^c^ | |  |  |  |
|  | Worse | 27 | 10.33 (13.54) | 0.0040 |
|  | Stable/No Change | 31 | –0.35 (13.47) |  |
|  | Minimal Improvement | 53 | –9.83 (12.20) |  |
|  | Much Improvement | 45 | –21.98 (17.43 |  |

^a^ Change in CGSQ scores from screening to Week 3 ^b^ ANOVA with linear trend ^c^ Patient categories defined by ADHD-RS-IV score change between screening and Week 3, with negative values representing improvement; Worse = ADHD-RS-IV change > 3 points; Stable/no change = ADHD-RS-IV –3 to 3 score change; Minimal improvement = –4 to –19 score change; Much improvement = –20 to –54 score change
ADHD-AM-RS, attention-deficit/hyperactivity disorder rating scale for 6:00 AM to 9:00 AM only; ADHD-RS-IV, attention-deficit/hyperactivity disorder rating scale-IV; CGI-I, Clinician Global Impression–Improvement; CGI-P, Conners’ Global Index–Parent; CGSQ, Caregiver Strain Questionnaire; SD, standard deviation
